# Algorithmic Accountability for Record Linkage

**DOI:** 10.23889/ijpds.v9i5.2854

**Published:** 2024-09-18

**Authors:** Emily Wiegand, Aya Liu, Sabrina Sedovic

**Affiliations:** 1 NORC at the University of Chicago; 2 Chapin Hall at the University of Chicago

This session will examine record linkage approaches through the lens of principles for algorithmic accountability: responsibility, explainability, accuracy, auditability, and fairness.[1] It is easy to hypothesize cases where linkage algorithms could have disparate impacts, such as linking names more or less easily depending on their cultural background. How can researchers use careful linkage design and sensitivity testing to identify and, where necessary, mediate these impacts? What does it look like for record linkage processes to be explainable and auditable—and how can researchers invite engagement from broader groups of stakeholders in considering linkage methods?

We will present on our experiences identifying and addressing these challenges in the context of linking state and local public sector administrative data sources. These data sources, which generally include populations engaging with particular programs or systems, have especially variable data quality and are often marred by structural biases. Our approach to linking these datasets emphasizes integrating policy and data context, interrogating linkage decisions for iterative improvement, and collaborating with a wide range of stakeholders for equitable, accountable data use. We will demonstrate examples of how linkage decisions and results have varied across contexts.

[1] Diakopoulos, N., Friedler, S., Arenas, M., Barocas, S., Hay, M., Howe, B., Jagadish, H.V., Unsworth, K., Sahuguet, A., Venkatasubramanian, S., Wilson, C., Yu, C., and Zevenbergen, B. (2016). Principles for Accountable Algorithms and a Social Impact Statement for Algorithms. Fairness, Accountability, and Transparency in Machine Learning. https://www.fatml.org/resources/principles-for-accountable-algorithms.

